# Fuels substitution possibilities, environment and the technological progress in Bangladesh’s transport sector

**DOI:** 10.1016/j.heliyon.2023.e13300

**Published:** 2023-01-29

**Authors:** Muhammad Yousaf Raza

**Affiliations:** School of Economics, Shandong Technology and Business University, Yantai, Shandong, 255000, China

**Keywords:** Transport sector, Factor’s substitution, Translog production function, Bangladesh

## Abstract

The transport sector is a key engine of Bangladesh’s quick oil demand growth. It accounted for 64.4% of overall Bangladesh oil consumption in 2019 and is, therefore, a third contributor to CO_2_ emissions and related pollutants. The substitutability of energy and non-energy factors is the key issue in framing and planning energy policies. Therefore, we determine a translog production function for the transport sector, including inputs labor, capital and energy. The research analyzes factor output and substitution possibilities from 1990 to 2019. Outcomes show (a) labor output elasticity is higher, followed by energy and capital. (b) All the substituting factors are rising return to scale, with relatively high substitution (around 1.63–2.05, 1.05–1.06, 0.77–0.92) between capital-labor, capital-energy and labor-energy, which proposes that the substitution between capital-labor and capital-energy could be attained through updating technology. Therefore, by giving maximum capital to the transport sector, appropriate energy-conserving technology could be maximally encouraged, and capital-energy substitutability would have better results in the future. (c) Though, technical progress is calculated to be between 0.009 and 0.14 between the various inputs. The input labor-energy is quicker substitutes with their relative difference in technological progress, while capital also presents proof of convergence. By assigning additional capital to the transport sector, energy-saving technologies could be enhanced and CO_2_ emissions reduction could be achieved. Finally, advancement in capital and skilled labor and, thus, substitution between energy-labor and the transition of labor-capital can be achieved.

## 1 Introduction

The transport sector is imperative in a market-oriented economy and plays an important role in influencing income scale worldwide [1]. Although, transportation is also liable for a huge proportion of energy consumption and it is still responsible for 24% of direct carbon dioxide emissions (CO_2es_) from fuel combustion (International Energy Agency (IEA) [2]). Also, road vehicles, including cars, buses, and 2–3 wheelers, account for almost 3-quarters of transport CO_2es_, and CO_2es_ from aviation and shipping to rise, which presents the need for worldwide policies. It has become one of the large energy-consuming sectors that consider energy conservation and global warming because of its large dependence on oil and inadequate reliable substitutive fuels [3]. The transport sector indicates among the most important sectors, including human doings globally and creating a relationship between humans, regions, and economic ways [4]. As per the IEA [5], global transportation’s energy use and CO_2es_ will rise by almost 50% by 2030 and by over 80% by 2050. The rapid expansion of road vehicles, especially private vehicles, has ensured in rising of the overall oil energy consumption by 2638.60 million tons of oil equivalent (Mtoe) in 2019 [2], which has broadly accepted as the main factor impacting future oil availability prices, and a significant contribution to world CO_2es_. Thus, the road sector leads the CO_2es_ by 11.9% by road transport, aviation by 1.9%, and shipping by 1.7%, 4.3% by navigations, rail by 1.8%, and rail by 0.4% and pipelines by 0.3% [6].

Geographically, Bangladesh has numerous opportunities, such as delta plain, predominately forest, coastal line of 710 km, airline routes, ports, and roads for export and import with bordering countries (i.e., India and Myanmar) proposing the most cost-effective and productive transit ways. These lines link central Asian countries and give a feasible and economical route to neighboring countries, such as China, Pakistan, Iran, and Afghanistan. Thus, regional and international transport networking with Bangladesh and other communication system enhancement is necessary. As per the Bangladesh Bureau of Statistics [7], the transport sector has added 11.04% to the country’s gross domestic product (GDP); therefore, it is necessary to make a developed and efficient transport and communication system that will link national and international roads as well as with Information and Communication Technologies (ICT) Networks. Padma Bridge, Metro-rail, rapid bus transit, Dhaka elevated express, and a few other mega projects are being employed for future and mutual linkages.

The total road length in Bangladesh is 22,418.95 km as of the year 2020 [8]. In addition, to operate the Railway as an environmentally friendly, safe, affordable, and reliable means of transport, various development programs have been undertaken and implemented on the total railway length of 3018.88 km. Also, several estimations have been initiated for the maintenance and development of various river routes to save watercraft movement, develop river ports to carry containers safely, etc. The Chattogram seaport is the main port where almost 92% of international trade is made. National flagship carrier Biman Bangladesh Airlines conducts 19 international flights and seven national flights on different routes, carrying 22,651 tons of cargo. Keeping stability with targets of European and Asian countries, the government of Bangladesh has taken various policy initiatives based on information technology using e-government and e-commerce services, as given in Table 1. These policies expect the transport sector to meet its conditional CO_2es_ lessening target below 24% by 2030 [9].

**Table 1:** Conditional targets and policy measures of Bangladesh’s transport sectors [9].

No.	Transport related policies
1	Modernizing the transport system, lessening consumption and enhancing lower carbon transport ways.
2	Demand management of road transport could be transported by congestion charging, encouraging a modal shift from private transport to public transport.
3	The NDC accepts a 25% by 2030 reduction in passenger-kilometer (km) travelled by road, with a successive rise in passenger-km for rail. 25% shift from the road to the rail sector will bring a major transformation in the rail sector. This will need heavy investment for the rail sector’s reliability by purchasing new engines, passenger rolling stock, new lines, new railway contracts, and updating rail traffic signals.
4	The NDC assumes a 15% reduction in fuel consumed by road transport per km travelled. This will impact locomotive efficiency, reduce emissions, train drivers, electronic signals, green freight, and old vehicle scrappage.
5	Consider the scope for making Nationally Appropriate Mitigation Actions in the transport sector to attract international support for their implementation.
6	Build up vehicle maintenance/service industry, ICT support on public transport.

The transport sector, the third-largest CO_2_ emitting sector after power and industries [10], has impacted the environment, society, trade, services, and natural resources. Ecological variation, social issues, and transport CO_2es_ are challenging for emerging nations like Bangladesh, for example in China, the transportation sector has proven by employing social and environmental targets in reducing pollution, accidents, and road destruction [11,12].

**Fig. 1:**
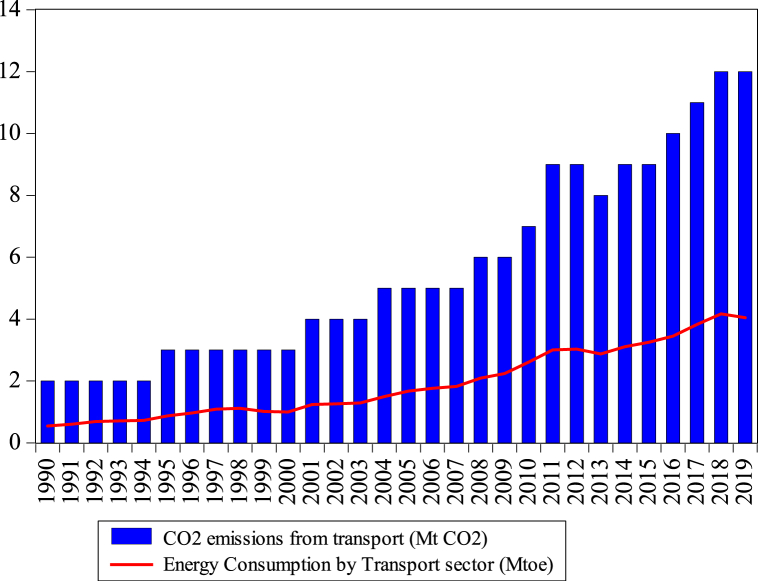
Consumption of energy and CO_2es_ from the transport sector of Bangladesh from 1990 to 2019. *Source:* IEA [5].

**Fig. 2:**
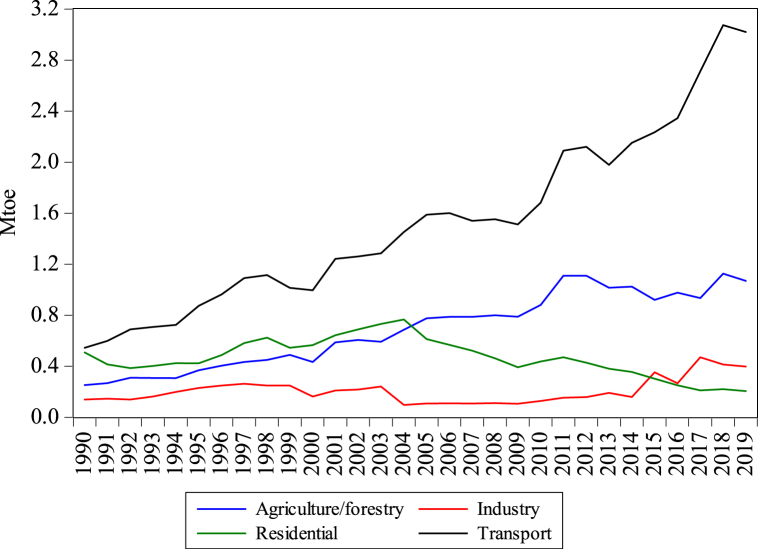
Sectorial oil consumption of Bangladesh from 1990 to 2019. *Source:* IEA [5].

**Fig. 3:**
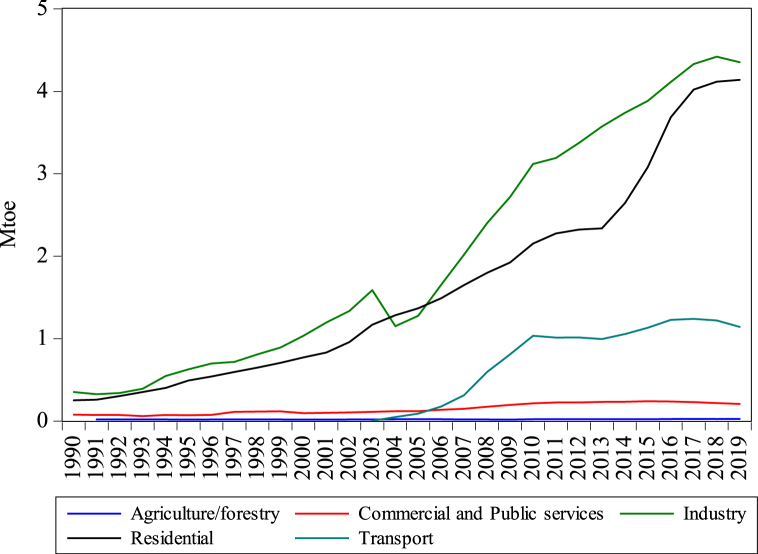
Sector-wise natural gas consumption of Bangladesh from 1990 to 2019. *Source:* IEA [5].

Fig. 1 presents the rising energy use and subsequent CO_2es_ from Bangladesh’s transport sector. Fig. 2 shows that oil is the record consumed energy source in the transport sector reaching 0.54–3.02 Mtoe from 1990 to 2019, showing the highest oil consumption of all other sectors. The oil used in agriculture, residential, and industry reaches 1.07, 0.204, and 0.397 Mtoe in 2019. Oil consumption in the transport sector raised by 4.54%, which has become the key oil-intense sector, even industrial, agriculture and residential divisions are left behind. Natural gas consumption in different sectors, such as industrial, residential, transport, commercial, and agriculture, is presented in Fig. 3. As presented in Fig. 3, the consumption of natural gas in the transport sector is 1.14 Mtoe, which started consuming in 2003. Utilizing gas in the transportation division is not higher than industrial and residential sectors, while agriculture and commercial sectors have lower gas consumption than the transport sector. Obviously, the annual growth rate of the transport sector is rising by 6.398%.

In the context of scientific research, the transport sector is an engine of economic and social development, created 8222 Mt CO_2es_ on the global scale in 2019 and has been the second biggest carbon emission source [62]. Though, in 2015, the top ten CO_2_-emitting countries of the world were China, US, India, Japan, Iran, South Korea, Canada, and Saudi Arabia, respectively, whose pollution emissions added to the world two-thirds [63]. In 2018, a few countries, such as China, India and the US added 85% of the total emissions, while Germany, Japan, Mexico, France and the United Kingdom lessened the CO_2es_. This is because different countries built policies under technological development and fuel substitution to declining fossil fuel consumption to minimize their share of global emissions [64,72]. For example, Lin and Raza [38] and Raza and Tang [65] examined the fuel substitution possibilities for Pakistan’s transport sector, which is directly or indirectly connected to the economy, social and scientific effects. Thus, to combat climate change and sustain the economy, the Intergovernmental Panel on Climate Change (IPCC) [66] and Intended Nationally Determined Contributions at COP-21 in Paris in 2015 are the best ways to control CO_2es_. To attain this goal, the Bangladesh government should make more efforts (i.e., policies, emissions characteristics and transmit channel) to concentrate on energy substitution and CO_2_ reduction.

The motivation and innovation of this study are as follows: first, as per the energy and non-energy factors, it is necessary to analyze the effectual output of the transport sector of Bangladesh. The factors and their substitution are important for transport planning, energy consumption, technical progress, and CO_2es_ lessening in Bangladesh, which is in line with Raza and Hassan [67]. Concerning current literature, few researches are found that especially studied the causality relationship between energy and CO_2es_ and natural gas consumption in Bangladesh’s various sectors (for instance, [22,23,25,59,67]). Even based on sectors and country-wise studies, they do not concentrate on energy substitution, CO_2es_ reduction scenarios and technological progress, especially in the transport sector. Second, very little proof related to the factor’s substitution in the transport sector has been seen; therefore, the energy supply employing production methods may be more suitable if the substitution elasticities are chosen. Third, the research uses a trans-log production function to analyze and measure the substitution between energy (i.e., oil and gas) and non-energy (labor and capital) factors for Bangladesh’s transport sector to give policy suggestions and figure out real transport structures for the present and future. According to study objectives, the current study examines the degree to which energy and non-energy inputs are useful and could be substituted to guarantee policies to get maximum economic growth with pollution reduction, and energy security. According to Christensen et al. [44], the trans-log production method is the right way to estimate the factor’s output elasticity, substitution and technical progress. It has more advantages, such as (i) this method is a quadratic reaction surface model, (b) shows the perfect substitution and competition between the factors, (c) avoids the imposition of the assumption of perfect substitution, (d) the existence of quadratic terms permits for a non-linear association between factor inputs [68], and (e) it reveals the interface regressors to show variables in the function. Furthermore, a common technique in the literature of energy economics in estimating energy demand elasticities has been determined as the implication of the trans-log cost function, which requires data on input costs, for instance, rents, wages and energy prices. However, this information is not reachable to authors during the examined period. Thus, the current study applies the trans-log production function to analyze the degree of output, substitution and technological progress between the estimated factors. Generally, three types of methods were used to check the energy and environment in various countries. (a) Logarithmic mean Divisia index to decompose the various factors due to its ease of use, interpretation and adaptation [59], (b) the econometric analysis based on the Environmental Kuznets Curve (EKC) [60] is employed to analyze the environmental quality and income along with U-shaped curve, presenting that it is inappropriate for an individual sector [61]. But, the trans-log production function is advantageous for estimating substitutability between input factors and reflects the interface of inputs to discuss variables in the function [18]. Finally, past studies (excluding Bangladesh) focused on road transport based on old statistics (i.e., cars, heavy transport, taxi, buses, and train) and transport CO_2es_, but the current study is based on updated statistics and include all kinds of transportation (i.e., land transport, water transport, air transport, support transport services, and post and telecommunication) in Bangladesh and transport CO_2es_ policies linked to NDC [9]. This originality has produced a gap between the past studies.

Further part of the current study is organized as: section 2 gives the literature review. Section 3 consists of econometric methods. Section 4 provides the data processes. Section 5 provides the results and discussion, while the conclusion and policy suggestions are given in section 6.

## 2 Background and literature review

**Fig. 4:**
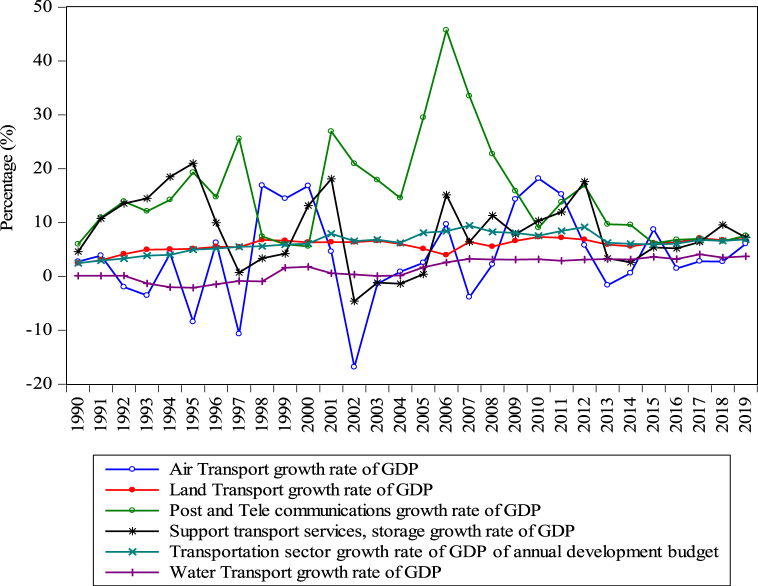
Bangladesh’s annual economic growth in the transportation sector (%) from 1990 to 2019. *Source:* calculated by the author [8].

During the last two decades, Bangladesh’s transport sector has been one of the highest-priority investment sectors for the Government and related agencies [13]. During the period, more than $50 billion was invested in the country’s transport sector, which significantly impacted the GDP. The post and telecommunication sector, particularly attracted the maximum share in the annual GDP growth rate (reached 6.9%) during the 2019 budget, support transport services contributed by 7.2%, air transport by 6.04%, land transport by 6.93%, water transport by 3.72% during 2019, which are rising over the period, as shown in Fig. 4. The current annual budget of the transportation sector shows a consistent rise during the period, such as 6.88% of the entire transport sector. Consequently, the GDP growth rate presents that the transportation sector and the number of road vehicles, airways and waterways showed a rapidly rising trend [8].

Among the various transport sources, road transport is taken to the fortitude of Bangladesh transport. As per the Bangladesh Economic Review [8], various roads, such as national highway 3906 km, regional highway 4767 km and Zilla road 13,423 km, are available under the roads and highways department. Similarly, the railway network spread about 3018.88 km around 43 districts, covering almost all the important places in Bangladesh. The ministry of communication has further undertaken 37 approved projects to implement in 2021 in the remaining districts. Overall, inland freight carries 1075.14 tons of km (million) and 14334.76 passenger-km (million) travel through roads. Therefore, the majority of vehicles use oil and natural gas. The transport sector is the largest oil user in Bangladesh [2,14]. This is due to the continuous rise of the population, and the volume of vehicles during the previous decade grew by a 438.44 quantum index of industrial production in 2018.

As transportation, especially the road sector is the very astounding contributor to ecological variation in Bangladesh. As per The World Bank [15] report, the air quality in the urban areas of Bangladesh is perilous due to the rise in transportation. For this, the government has bridge projects to support demonstrative physical investments in traffic management that add to enhancement in air quality, technical capacity and institutional reforms before assisting a more practical and determined urban transport initiative. In addition, the main objective is to advance the railway system and urbanization and improve the rail and transport services on a priority basis. As per Bangladesh Economic Review [8], Bangladesh’s 8th Five-year plan and perspective plan, Vision-2021 and a higher budget have been added for the development of the railway than in the past. In this case, 230 projects with a cost of 5,53,662 crore Tk. are added to implement six stages from July 2016 to June 2045. These targets must have a huge level of skilled labor, human resource, energy, and capital in Bangladesh. To save energy and the environment in the transportation sector, the country requires huge investments in capital and advanced technologies; thus, the present condition encourages us to investigate the CO_2es_ from the transport sector of Bangladesh.

Regarding the chances and challenges, present output and future viewpoint for transportation expansion; Bangladesh transport acquires worldwide values from the outlook for efficiency, atmosphere and energy conservation. Bangladesh has signed two World Bank-supported projects to support lessening energy deficiency and encourage the transport division, such as the Air Quality Management Project (AQMP) and Learning and Innovation Loan (LIL). The objective of these projects is to develop and implement actions that were multi-sectorial, and that presented how to widely report air quality and urban transport issues [15]. From the economic perspective projects, such as Clean Air and Sustainable Environment (CASE) will provide 11.03 times as many economic benefits with an economic net value of US$892.55 million. Except it, World Bank has asked the Government to enhance air excellence on an urgent basis, as the air quality in the world is getting worse. Bangladesh finds itself in a difficult state and; thus, it has to control its upcoming energy policy rightly. As the largest oil-consuming and second CO_2_-emitting sector after the industrial sector, Bangladesh can play an imperative part in this background. The elasticity of substitution between energy and non-energy factor inputs is seriously imperative for energy policy in various sectors, analysis and planning. The implication of energy-conserving technologies in terms of production operation might be described by the substitutability of capital for energy [16]. Analyzing the substitutability between energy (total energy consumption by the transport sector) and non-energy (capital and labor) factors is also crucial for various output, energy and pollution issues. These matters can be further calculated as the subjects of trading carbon payments, reprocessing energy-related tax income to lessen non-energy factor tax and gradually increasing fuel taxes [12,17]. Since variations shall not be carried-out should not come at a growth cost; thus, the country wants a subtle complimentary act to forward in its plan to grow transport’s productivity and enlighten energy security and ecological sustainability. Furthermore, it is necessary to discuss that Bangladesh’s transport sector is currently facing numerous issues, such as huge consumption of fossil fuel, infrastructure, CO_2es_ and labor-intensive for maximum output. It is, therefore, the substitutability between labor, capital and energy consumption imperative to energy policy from a future perspective. The key objectives explore to see the transport energy, and CO_2es_ and provide policies to attain maximum economic growth, energy substitutability and natural maintainability.

Since energy is one of the key production factors, energy and non-energy inputs are set into production as per a certain portion and can substitute for one another to certain level. Under the principle of substitutability, it is probable to achieve the dual goals of economic progress and energy conservation by shifting the combination of energy and non-energy inputs. Hence, there are four main points that should analyze in the transport sector. (i) To what extent can other inputs substitute for energy, for example, fossil fuels to renewable energy or low-carbon emitting fuels?; (ii) what are the impacts of substitutability outcomes on the transport sector’s development and energy conservation?; (iii) what is energy-saving and CO_2es_ reduction in Bangladesh’s transport sector in the current scenarios from 2017 to 2019?, and (iv) in the scientific and societal community, what is the relative difference in the technological progress of energy and non-energy factors during the analyzed during 1990–2019? Thus, to achieve future demand and supply, the government should focus on energy, non-energy, inter-factor, and inter-fuel substitution levels. In specific, the results of future forecasts for energy needs are consistent when the demand models consider substitution elasticity [58]. Finally, the key goal of this estimation is to look at the transport-related CO_2es_ and provide policy suggestions to obtain high economic growth, energy substitution, and natural maintainability.

A large number of research studies have been carried out in Bangladesh with various econometric techniques in analyzing the energy and economic relationship. For example, Bala [19] employed the LEAP method to analyze the energy and CO_2es_ relationship; Mondal et al. [20] used the MARKAL method to analyze the energy, technology transfer and CO_2es_ relationship; Miah et al. [21] employed the EKC hypothesis to estimate economic and environmental relationship; Jahangir et al. [22] applied the cointegration method to find the causal relationship between energy and CO_2es_; Shahbaz et al. [23] employed an ARDL method to investigate the relationship between financial development, energy consumption and CO_2es_; Habib and Chungpaibulpatana [24] used scenario method, and Karmaker et al. [25] applied Hybrid optimization of multiple energy resources to examine the CO_2es_ from various fuels. Moreover, economic growth speed up the process of industrialization, such as agriculture, transport, manufacturing and mining [70]; Usman et al. [71] analyzed the relationship between technological innovation, energy and natural resources from 1990 to 2018 and found that technical innovation and human capital can bring newness in the firms and establish the sustainable development; Balsalobre-Lorente et al. [73] estimated EKC and pollution heaven hypothesis in PIIGS countries and found that urbanization applies huge pressure on environmental quality while renewable energy declines the pollution, and Usman and Hammar [74] investigated the association between technological innovation, renewable energy and carbon footprint for Asia-Pacific countries from 1990 to 2017 using the STRIPAT model. They found that technical, economic growth and population growth impact the environment in the long-run process. The literature on energy and related factors substitution in Bangladesh is very little; especially the possibility of switching between energy and non-energy factors in the transportation sector has been neglected by many Bangladeshi scholars concerned with the energy economy. Thus, the literature on approximations of inter-factor and inter-fuel substitution potentials of energy demand for Bangladesh is almost absent.

Measuring the factor substitutability is necessary for estimating energy and ecological policies. There are numerous measuring techniques for energy substitution elasticity. Thompson [26] examined the applied elasticity theory of substitution on translog cost and translog production functions, whose economic properties and elastic practices are useful for developing awareness and applying energy substitutability. A detailed survey of classical elasticities of substitution was conducted based on the translog cost method by Frondel [17] that involves Morishima, McFadden, Allen, and cross-price elasticities as compulsory and imperative elements. As per the literature related to energy substitution, few researchers supposed that substitution elasticities are constant, and they applied constant elasticity of substitution (CES) in their research, for instance, Su et al. [27] employed a two-stage CES method to measure the substitution elasticities between capital, labor and energy during 1953–2006; Zha and Zhou [28] combined the CES along with translog production and cost function. They found the substitution elasticity between energy and related inputs for the industrial division of China during 1994–2008; Zha et al. [29] formally employed the CES function to investigate the technical bias in China’s industrial sector during 1981–2017. They found that most metal-intensive industries' capital is nested with labor. Though, the CES assumptions may be too hard. There is no motive to consider that substitution elasticity is a constant; indeed, it might fluctuate over the period. Because of the constraints of the CES function, the translog production function usage is a more general method of factor substitution elasticity and can be easily interpreted as 2nd-order Taylor estimation for arbitrary function. Therefore, the CES function is merely a particular example of the translog production method.

In general, there exist ‘3’ kinds of relationships between energy and non-energy inputs, i.e., complementarity, substitutability and uncertainty. Precisely, keeping economic production constant, the rise in non-energy inputs will affect energy consumption to perform ‘3’ various roles, i.e., decline, rise and uncertainty. For instance, in the related literature, Adetutu [30] examined the substitution between energy output and capital for ‘4’ OPEC countries and found that there is capital-energy substitution in Saudi Arabia and Algeria, but complements in Venezuela and Iran. Burki [31] observed adaptation of substitution and complementarity between capital-energy in the manufacturing division of Pakistan. Liu et al. [32] analyzed factor substitutability and carbon intensity in China’s heavy industry. They found that almost 45.77% variation in carbon intensity is attributed to labor-energy and capital-energy substitution. Kim and Heo [33] examined the substitutability between energy-capital in ‘10’ manufacturing industries in OECD nations and found that there is a substitution between energy and capital. Lin and Liu [34] examined the energy switch impact in the machinery sector of China and developed that there is a significant substitution between energy-capital and energy-labor. Linked with the capital-energy substitution, that of energy capital is dominated. As per the above viewpoint, asymmetric substitutability is sensible for various sectors, for capital is highly costly than energy in those republics.

The translog production function being the second-order differential equation at a definite point where functional forms charge no boundaries or restriction on the production process has further advantages: (a) avoids utilizing the factor prices' data (b) generally applied to study inter-factor substitutability and (c) have no conditions on first or second differentiation at the point of estimation. For instance, Raza et al. [35] analyzed the potential for substitution between labor, capital and energy for Pakistan’s chemical sector. They analyzed that the substitution elasticity between capital-energy and labor-energy is significant and substitutes for one another. Smyth et al. [18] analyzed the substitutability between energy and non-energy factor in the steel industry of China. They found that there is a replacement between capital-energy and energy-labor. Similarly, various studies were conducted in a series in various regions, sectors and countries to investigate the productivity and related policy measures, such as Wesseh et al. [36] for Liberia’s energy economy; Lin and Xie [37] for the transport division of China; Lin and Raza [38] for Pakistan’s agriculture sector; Guidolin and Guseo [39] for German energy transition; Lin and Atsagli [40] for South Africa energy substitution; Suh [41] for United States biomass energy; Zhao et al. [42] for China’s various energy substitution targets in 2020 and 2030, and Lin and Tian [43] for China’s light industry.

From Bangladesh’s perspective, very few studies have been found on the subject (i.e., energy, economy and pollution) based on various models, as discussed above. Moreover, the literature review earlier has given studies of energy substitution based on various types of methods. Though, we did not find the literature on energy and non-energy factor substitution in the transport sector of Bangladesh. The current study assessed the energy substitution, including labor, capital and transport energy consumption using translog production function during 1990–2019. Thus, the study suggests policies for enhancing energy policies, energy conservation and carbon reduction from the transportation sector. Consequently, this study may make up for the gap based on the corrected formula.

## 3 Econometric method for estimation

### 3.1 Translog production model and output elasticity

The employed function (translog production function) is a kind of quadratic response surface model in the context of the structure. This function can be utilized to investigate the interface between input factors in the production process. In addition, this method has both linear and quadratic terms with the capability of employing more than ‘2’ inputs, and it can be estimated to employ the 2nd order Taylor series [44]. In the current research, employed labor, capital stock and energy consumption are considered as input factors to improve a translog production function for Bangladesh’s transport sector. As per the model description, out elasticity and elasticity of substitution are investigated in which converted turnover volume is considered as output (Y) of the transport sector. Thus, based on the Taylor series, the general functional form is as follows:

(1) $$\begin{array}{l} \ln\;Y_{t}=\alpha_{0}+\alpha_{K}\;\ln\;K_{t}+\alpha_{L}\;\ln\;L_{t}+\alpha_{TEC}\;\ln\;TEC_{t}+\alpha_{K.L}\;\ln\;K_{t}\times \ln\;L_{t}+\alpha_{K.TEC}\;\ln\;K_{t}\times \ln\;TEC_{t}+\\ \alpha_{L.TEC}\;\ln\;L_{t}\times \ln\;TEC_{t}+\alpha_{K.K}{\left(\ln\;K\right)}^{2}+\alpha_{L.L}{\left(\ln\;L\right)}^{2}+\alpha_{TEC.TEC}{\left(\ln\;TEC\right)}^{2} \end{array}$$

As per the above expression in Eq. (1), $Y_{t}$ shows the output of Bangladesh’s transport sector; $K_{t}$, $L_{t}$ and ${TEC}_{t}$ are the inputs of capital, labor and transport energy consumption, respectively. ∝ is the parameter to be estimated. t is the time index.

As per Eq. (1), characterizing the economic region of a linear homogenous production function, it is necessary to calculate the output elasticity of each factor of the K, L and TEC inputs. These estimations are as:

(2) $$\varphi_{Kt}=\left(\frac{d\;\ln\;Y_{t}}{d\;\ln\;K_{t}}\right)=\alpha_{K}+\alpha_{K.L}\;\ln\;L_{t}+\alpha_{K.TEC}\;\ln\;TEC_{t}+2\alpha_{K.K}\;\ln\;K_{t}⊳0$$

(3) $$\varphi_{Lt}=\left(\frac{d\;\ln\;Y_{t}}{d\;\ln\;L_{t}}\right)=\alpha_{L}+\alpha_{K.L}\;\ln\;K_{t}+\alpha_{L.TEC}\;\ln\;TEC_{t}+2\alpha_{L.L}\;\ln\;L_{t}⊳0$$

(4) $$\varphi_{TECt}=\left(\frac{d\;\ln\;Y_{t}}{d\;\ln\;TEC_{t}}\right)=\alpha_{TEC}+\alpha_{K.TEC}\;\ln\;K_{t}+\alpha_{L.TEC}\;\ln\;L_{t}+2\alpha_{TEC.TEC}\;\ln\;TEC_{t}⊳0$$

### 3.2 Factor’s elasticity of substitution

Multifactor substitutability of various factors using the translog production function is an addition to the CES or Cobb-Douglas function approach. The principal enhancement is that it permits the substitution elasticity to change with the percent of input factors, while the substitution elasticity between various pairs of inputs provides an imperative benefit. As substitution elasticity is a primary indicator to analyze the association between pairs of input factors [45], which can be defined as: it is the % change of the ratio in inputs to a % change of the ratio in the marginal rate of technical substitution. For instance, Lerner [46] described that the intuitive meaning of substitutability is the curvature of isoquant [47]. The elasticity of substitution changes from zero to infinite, and it illustrates how hard it is to substitute from one input to another input, which can be estimated as:

(5) $$\sigma_{ij}=\frac{\%\Delta \left(\frac{X_{it}}{X_{jt}}\right)}{\%\Delta \left(\frac{P_{jt}}{P_{it}}\right)}$$

It can be noted that “ij” is the pair of factors, i.e., K.L, K.TEC and L.TEC. Given the assumption that firms in the Bangladesh transport industry are cost-minimizing agents, Eq. (5) can be re-written as:

(6) $$\sigma_{ij}=\frac{\%\Delta \left(\frac{X_{it}}{X_{jt}}\right)}{\%\Delta \left(\frac{MP_{jt}}{MP_{it}}\right)}=\frac{d\left(\frac{X_{it}}{X_{jt}}\right)}{d\left(\frac{MP_{jt}}{MP_{it}}\right)}\times \left(\frac{\left(\frac{MP_{jt}}{MP_{it}}\right)}{\left(\frac{X_{it}}{X_{jt}}\right)}\right)$$

From Eq. (6), the elasticity of the substitution final formula based on pair of factors will become:

(7) $$\sigma_{ij}={\left[1+\frac{-\alpha_{ij}+\left(\frac{\varphi_{i}}{\varphi_{j}}\right)\times \alpha_{jj}}{-\varphi_{i}+\varphi_{j}}\right]}^{-1}$$

As per Eq. (7), the elasticity of substitution between capita-labor, capital-energy consumption and labor-energy consumption in Bangladesh can be estimated as:

(8) $$\sigma_{K.L}=\left|1+\right|-\alpha_{K.L}+\frac{\varphi K}{\varphi L}\times \alpha_{L.L}{\left|\times {\left(-\varphi K+\varphi L\right)}^{-1}\right|}^{-1}$$

(9) $$\sigma_{K.TEC}=\left|1+\right|-\alpha_{K.TEC}+\frac{\varphi K}{\varphi TEC}\times \alpha_{TEC.TEC}{\left|\times {\left(-\varphi K+\varphi TEC\right)}^{-1}\right|}^{-1}$$

(10) $$\sigma_{L.TEC}=\left|1+\right|-\alpha_{L.TEC}+\frac{\varphi L}{\varphi TEC}\times \alpha_{TEC.TEC}{\left|\times {\left(-\varphi L+\varphi TEC\right)}^{-1}\right|}^{-1}$$

From Eqs. (8), (9), (10), the $\sigma_{K.L}$, $\sigma_{K.TEC}$ and $\sigma_{L.TEC}$ present inter-factor and inter-fuel substitution elasticities between capital-labor, capital-transport energy consumption and labor-transport energy consumption. Positive outcomes of each pair indicate that the inputs are substituting, while negative outcomes show that inputs imply complements.

### 3.3 Ridge regression

The model could suffer from multicollinearity issues as a result of the interaction and squared terms of the input variables in Eq. (1). It is the phenomenon in which ‘2’ or more than ‘2’ independent variables in the multiple regression models are extremely interrelated. Thus, violating the key required conditions for Ordinary Least Square (OLS) to be unbiased. The coefficient measures for the design matrix ‘X’ have a proper linear dependence; thus, the matrix ${{(X}^{T}X)}^{-1}$ $X^{T}y$ becomes close to singular. As per Girod et al. [48], the OLS measure becomes very sensitive to random errors in the observed response ‘y’ because of high variance ($\sigma^{2}$). The matrix values can be estimated as shown in Eq. (11).

(11) $$\hat{\beta }={\left(X^{T}X\right)}^{-1}X^{T}y$$

This condition of multicollinearity can grow, for instance, when statistics are composed without an experimental pattern. To answer this issue, the ridge regression method was adopted, which was proposed by Hoerl and Kennard [49] and Hoerl and Kennard [50] for measurement, instead of the traditional OLS. Ridge regression deals with the problem by analyzing regression coefficients employing as shown in Eq. (12):

(12) $$\hat{\beta }={\left(X^{T}X+kI\right)}^{-1}X^{T}y$$

where “k” is known as the ridge parameter, which satisfies k ≥ 0, and I is known as the identity matrix. Generally, there is an optimal k-value for any issue; however, it is necessary to estimate the ridge solution for the threshold of “k” values. Lowering the k-values enhances the conditioning of the issue and lessens the $\sigma^{2}$ of the estimates, while for biased, the lowered $\sigma^{2}$ of “k” measures generally consequences in a lower mean square error when linked to OLS measures. As per the above literature, numerous techniques for getting the optimal “k” value have been projected. The current study employs the ridge trace technique, which is very common in the literature. In addition, the coefficients are calculated at different levels of “k,” which starts from [0–1]. The $\hat{\beta }$ coefficients are further designed regarding “k” values, and the optimum value is selected at the point where the $\hat{\beta }$ coefficients appear to be steady.

## 4 Data process and sources

In respect of the employed model, four major variables have been used in this study, including the output variable (Y) and inputs capital, labor, and energy consumption in Bangladesh’s transport sector. We analyzed these variables using the aggregate production function output with energy use, labor and capital variables that affect production throughout 1990–2019. In addition, Bangladesh is hugely dependent on fossil fuels, with little renewable energy for production, which could be a significant limitation. The physical capital, labor and energy consumption, we follow the standard growth model and expect that these factors positively impact economic development. For instance, Solow [69] in the neoclassical growth theory confirmed that the availability of capital and labor inputs in the production process; thus, increases production and economic activities. Finally, we assume energy consumption to leads economic growth. As per the growth hypothesis, energy is an important input in the production processes; thus, maximum energy use benefits economic growth [4,14]. Therefore, this study analyzes the energy and non-energy factors to measure the impact of transport infrastructure and innovation on current respective variables. The output and labor data are collected from Bangladesh Economic Review [8]. Capital data has been taken from World Development Indicators, while all the energy-related data was collected from International Energy Agency [2,10]. Data related to capital stock is not directly available in Bangladesh statistics, which is collected from World Bank indicators because many scholars have employed this kind of data for various sectors and regions, such as Chen [51] for 38 sub-industries of China and Lin and Raza [52] for the transport sector of Pakistan. The capital stock is stated as physical capital in the true sense. Output data is converted into Crore Taka; energy-related data is taken in Tera joule (Tj). The capital stock is taken in billions of US$ while labor is considered in percentage. So, the perpetual inventory method (PIM) is employed to measure the real capital stock of Bangladesh’s transport sector, which can be stated as follows in Eqs. (13), (14):

(13) $$K_{t}=I_{t}+\left(1-\delta_{t}\right)\times K_{t-1,}$$

(14) K0=It(g+δ)

where K_t_ is capital stock, K_t−1_ is the previous year’s capital stock, I_t_ is the current capital investment, and δ is the capital depreciation rate. K_0_ and I_0_ are the initial capital stock and capital investments. ‘g’ is the average growth rate of capital investment from 1990 to 2019. As per Bangladesh’s investment policies, a 5% depreciation rate is taken [53], which has been considered by many developing countries, such as Lin and Xie [37] for China, Lin and Raza [52] for Pakistan and Lin and Atsagli [40] for South Africa.

**Fig. 5:**
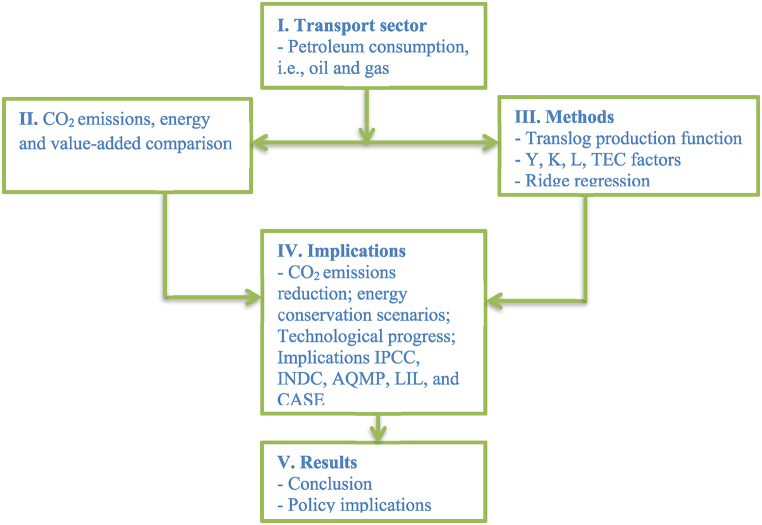
Pictorial form of the transport study under production function.

Actually, this method (PIM) was first suggested by Goldsmith [54] and was broadly applied in OECD nations. Practically, it contains a few variables to estimate the capital stock, for instance, fixed asset investment price, determination of depreciation rate and annual investment. However, in the present study, the annual series of capital extended to 2019, which has not been discussed before. The study chart based on the whole framework is provided in Fig. 5. The measurement process of this study can be divided into five parts, including sectorial information, pollution, methods, implications, and results and discussion. As per the statistical analysis, the I–O elasticities and technical progress of all the factors (i.e., labor, capital and energy consumption) are analyzed.

## 5 Results and discussion

### 5.1 Model output

Specified the number of measured parameters in the current translog production model, we started our analysis to estimate the multicollinearity issue in the data using the Kmenta [55] method. As per Kmenta, a simple multicollinearity degree is achieved by regressing every explanatory variable on the remaining explanatory variables. Since the equation has multiple quadratic terms, the model may suffer from a multicollinearity issue. For example, if the independent variable can be linearly explained by others, there is severe multicollinearity which might create problems to recognize arguments.

Thus, the multicollinearity problem can be verified through the Variance Inflation Factor (VIF). As per the general principle, if the VIF is larger than 10, the multicollinearity will be severe. If the VIF is higher, then the ridge regression technique could be employed to reduce it; thus, to confirm whether there is a multicollinearity issue in the data, we need to see the VIF of the individual independent variables in the model. As shown in Table 2, the VIFs of each independent variable are higher than 10, showing severe multicollinearity. It is, therefore, to solve this problem; we applied the ridge regression technique.

**Table 2:** VIF description of all independent variables.

Variables	VIF	1/VIF
lnL	257953.6	0.00000388
lnK	2439073	0.00000041
lnTEC	2511119	0.00000040
ln(L*L)	96716.75	0.00001034
ln(K*K)	242094.1	0.00000413
ln(TEC*TEC)	4840229	0.00000021
ln(K*L)	333142.3	0.00000300
ln(K*TEC)	6483474	0.00000015
ln(L*TEC)	1702702	0.00000059
VIF average	2100723	-

**Fig. 6:**
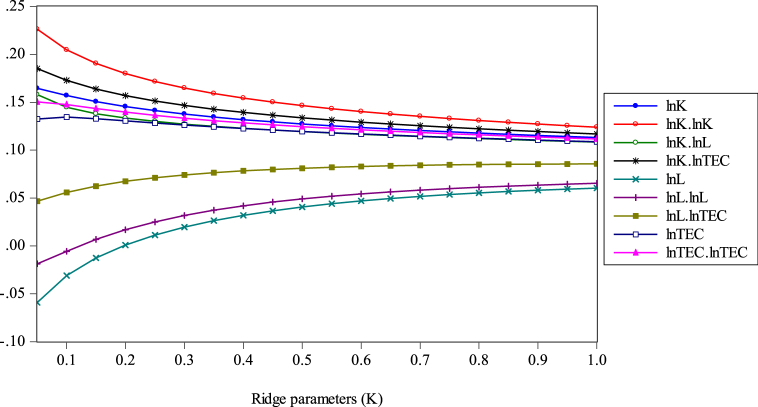
Ridge trace based on k-parameters.

**Fig. 7:**
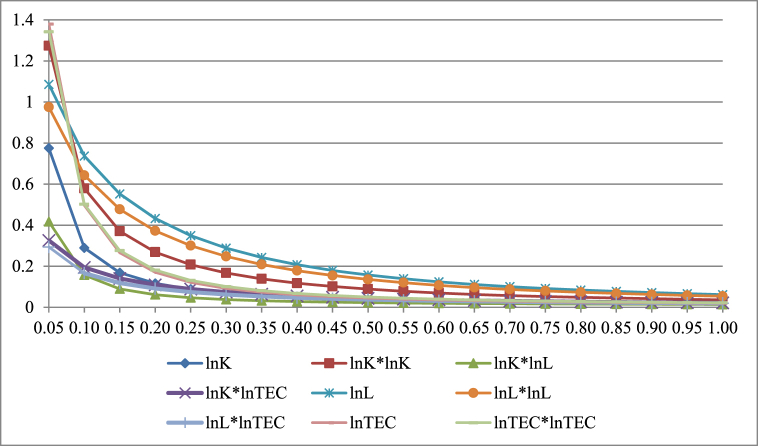
VIFs of the ridge regression variables. *Note:* Horizontal values are VIFs and vertical values are ridge parameters.

**Fig. 8:**
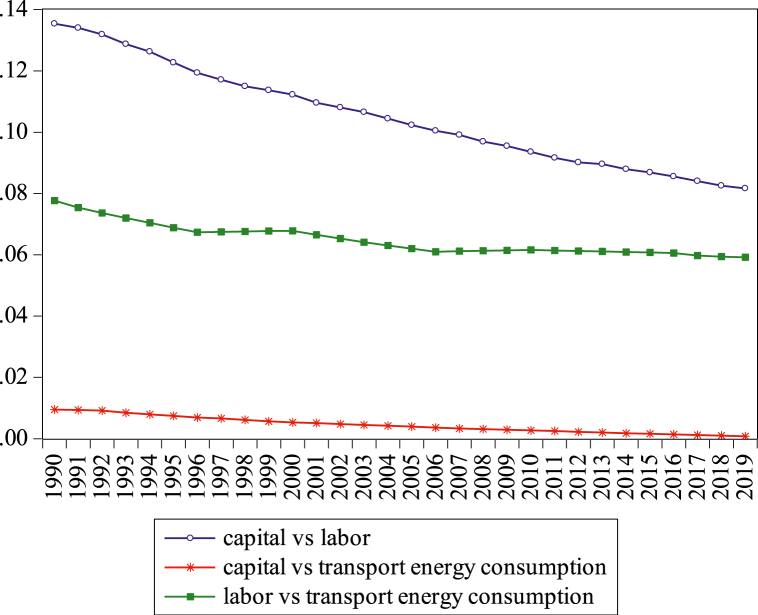
Difference in technical progress ($tp_{ij}$) among pairs of energy inputs from 1990 to 2019.

Generally, optimal statistics of ridge parameter (k) exist for the ridge regression model. The assumptions show: (1) if the k-value is very lower; the bias is relatively lower, and (2) if the k-value is higher, the bias is higher. Presently, our objective is to make the bias and VIF both as lower as possible; therefore, this is the dilemma. As per related econometric literature, a few scholars (i.e., Wesseh et al. [36]; Liu et al. [32]) projected numerous techniques for getting a better value for the k-parameter. They apply ridge regression to measure the parameters of the functions because of the multicollinearity in the data and found that all inputs are substitutes. We currently employ the ridge trace plot technique, which is widely employed. All the parameters used in this method are measured as per the k-values from “0” to “1”. The plotted ridge trace shows the best value at the specific point (k = 0.75) where all the coefficients appeared to stabilize. A huge multicollinearity issue leads us to move the measured coefficient from OLS-ridge regression. The ridge trace plot about the coefficient measures is given in Fig. 6. It is obvious in Fig. 6 that the ridge trace looks stable when k = 0.75. In addition, Fig. 8 presents that VIFs rapidly fall initially, and then become exceedingly gentle when k > 0.75. Conclusively, Fig. 6, Fig. 7 confirm that the k = 0.75 is suitable .

### 5.2 Ridge regression

After identifying the k-value, we measure the ridge regression. The relevant statistical analysis is presented in Table 3, which shows that ridge regression model outcomes are significant. All the analyzing indicators, i.e., standard error (SE), P-value (significant value), coefficient of determination (R-square), and coefficients (beta values), are together, indicating that the model is fit and reasonable. More significantly, either the current model is good or not relies on if the ridge regression has reduced the optimal multicollinearity issue and whether the investigated parameters are suitable or not. Obviously, we can see in Table 3 that all the statistics and related coefficient measures are ideal because of the lower standard error. Almost more than 95% of SE coefficients are lower than 5%. All the regression coefficients are optimistic about 0–0.13, which is in line with the economic reality. It is also obvious that all the multiple-influence items are positive and significant. These outcomes are close to the studies of Lin and Liu [34] and Lin and Xie [37] for the machinery and transport sector in China. They found that substitution between capital-energy and labor-energy is productive. This suggests that energy labor and capital could be attained by continuously improving technology. This presents that all inputs, including capital, labor and transport energy, present a rising return to scale. It is, therefore, all the selected parameters are suitable for the model equation.

**Table 3:** Outcomes of a ridge regression model.

Variables	Coefficients	Standard Error (S.E)	P-values	VIF
lnL	0.05361	0.03643	0.08968	0.09179
lnK	0.11907	0.02444	0.00062	0.02510
lnTEC	0.11342	0.02504	0.00096	0.02717
ln(K*L)	0.11307	0.02508	0.00099	0.01655
ln(K*TEC)	0.12379	0.02397	0.00043	0.02872
ln(L*TEC)	0.08455	0.02901	0.00972	0.02504
ln(L*L)	0.05980	0.03449	0.06058	0.07966
ln(K*K)	0.13293	0.02313	0.00022	0.05240
ln(TEC*TEC)	0.11710	0.02465	0.00072	0.03021
Ridge k	0.75			
R-square	0.9732			

### 5.3 Output elasticity and elasticity of substitution

As per the energy and non-energy inputs, we used Eqs. (2), (3), (4) to evaluate output elasticities. The output elasticities are provided in Table 4. As presented in Table 4, the output elasticity of labor and energy is the maximum and the output elasticity of capital is the minimum. During 1990–2019, all the factor’s output elasticities are growing each year; however, their rising rates are rather moderate. This presents that the effects of the factors are raising returns to scale is narrowing in Bangladesh’s transport sector. These outcomes are in line with Lin and Xie [37] who analyzed that all the energy and non-energy factors of China’s transport sector are rising and Raza and Tang [65] for Pakistan found that output elasticities are rising and contributing to the economic development. Moreover, they found that substitution between capital, labor and energy shows higher substitution. This proves that the factor’s substitutability exists. Furthermore, the output elasticity between labor and energy utilization is close to ‘1’, which concludes that when labor and energy increase by 1%, the income of the transport sector will rise by more than 1%. Besides, the production function raises the return to scale for energy and labor. Though, the output elasticity of capital is about 0.87, which presents that when capital grows by 1%, the productivity wills variate by 0.87%. Thus, it is obvious from Table 4; the production function is decreasing return to scale for capital. Consequently, the growth in Bangladesh’s output is susceptible to raising employment in the transport sector.

**Table 4:** Output elasticity of production factors from 1990 to 2019.

Period	$\emptyset_{Lt}$	$\emptyset_{Kt}$	$\emptyset_{TECt}$	Period	$\emptyset_{Lt}$	$\emptyset_{Kt}$	$\emptyset_{TECt}$
1990	0.807538	0.551473	0.754622	2005	0.987460	0.718718	0.876404
1991	0.826903	0.558465	0.763652	2006	1.000705	0.731174	0.883887
1992	0.844446	0.568014	0.775387	2007	1.000411	0.739177	0.887628
1993	0.860259	0.581288	0.782193	2008	1.002849	0.752644	0.898158
1994	0.875318	0.592510	0.788054	2009	1.003080	0.761843	0.904006
1995	0.894724	0.608911	0.804157	2010	1.005560	0.774606	0.915266
1996	0.912110	0.624883	0.815301	2011	1.011947	0.788305	0.926488
1997	0.913782	0.634788	0.824291	2012	1.015216	0.799273	0.930213
1998	0.913916	0.644351	0.827795	2013	1.016035	0.803353	0.928696
1999	0.911364	0.650104	0.824194	2014	1.020876	0.815546	0.936238
2000	0.910879	0.656666	0.824883	2015	1.024503	0.824240	0.941258
2001	0.929640	0.672257	0.842305	2016	1.028642	0.834999	0.947448
2002	0.942791	0.681406	0.847115	2017	1.041723	0.849051	0.957928
2003	0.956063	0.690994	0.852430	2018	1.048727	0.862320	0.966497
2004	0.972210	0.704663	0.865374	2019	1.051737	0.870199	0.967379
				**Average**	**0.957714**	**0.711541**	**0.868642**

The pairs of the substitution elasticity among capital-labor (K-L), capital-transport energy consumption (K-TEC) and labor-transport energy consumption (L-TEC) are provided in Table 5. Table 5 presents that there is a continuous rise among all the factors during 1990–2019. The trend among K-L and K-TEC gives the highest substitution elasticity, while L-TEC shows the minimum substitutability among input factors. It is clear in Table 5 that changes in substitution elasticity between input factors in Bangladesh’s transportation remain moderate. All the pairs of substitution conclude that: substitutability between all the pair’s measures tends to be optimistic, proposing that all the pairs, i.e., K-L, K-TEC and L-TEC are substitutes. (b) The substitutability between L-TEC is quite close to the substitution elasticity between K-TEC. Both of the pairs are around 0.92–1.06 in the current thirty years. These results are close to the studies of Lin and Raza [52] and Lin and Liu [34] for transport and industries. They used energy, capital and labor factors for the transport sector and find that output elasticities and pairs of inputs are rising in which labor-energy and capital-energy present maximum potential due to their substitutability. As our results conclude that the level of substitution association exists between K-TEC and L-TEC. The outcomes suggest that by growing energy input, capital and labor can be efficiently attained in the transport sector of Bangladesh. (c) The substitution elasticity between K-TEC is slightly high, about 1.05–1.06 in the present 30 years, showing a rather rising trend. The outcome proposes that the substitutability between K-TEC in Bangladesh’s transport sector is efficient. In addition, the growing trend of factor substitution shows a higher gap for forwarding energy supply deficiency with a higher contribution. Bangladesh’s transport sector (including both public and private) is the main consumer of petroleum products, consuming about 53% of the total sales of petroleum products, which is consistent with the International Institute for Sustainable Development [56] which analyzed that petrol and diesel are the main fuels for transportation, which can be substituted with other fuels, such as gas and renewables. The substitution between K-TEC must appreciate finding an efficient way of saving energy. (d) The elasticity of substitution between K-L is found to be the highest, about 1.63–2.05 from 1990 to 2019. As per the previous information on transport-related investment (i.e., various projects including Padma Bridge, Metro-rail, Bus Rapid Transit, Dhaka Elevated Expressway, and some other mega-projects) and labor input in the transport sector, we find that capital-labor input is rising quickly each year [8]. During 1990–2019, K-L inputs indicate an increasing annual trend of 9.35% and 1.65%. In fact, substitutability between K-L is unavoidably caused by technical development. This is because as the transportation sector grows, the technical enhancement releases some extra labor due to huge capital to attain numerous things that the country was supposed to be taken manually. As shown in Table 5, there is an increasing trend of K-L substitution with a significant result. This presents that there is still a significant substitution relationship between K-L with further technical enhancement in Bangladesh’s transport sector. Consequently, it is observed from Table 4, Table 5, the output elasticity shows a higher return concerning labor and energy, while substitutability between capital, energy and labor shows significant substitutability. This should not be astonishing since this is the real condition in Bangladesh’s economy, where there has been growing labor investment and comparatively fewer capital motives. Therefore, we have implied the scenarios based on growing capital and energy to check the country’s environmental situation.

**Table 5:** The elasticity of substitution between input factors from 1990 to 2019.

Period	$\sigma_{K.L}$	$\sigma_{K.TEC}$	$\sigma_{L.TEC}$	Period	$\sigma_{K.L}$	$\sigma_{K.TEC}$	$\sigma_{L.TEC}$
1990	1.631951	0.774179	1.055146	2005	1.742872	0.844424	1.076300
1991	1.614712	0.774549	1.059822	2006	1.750319	0.850604	1.078381
1992	1.608578	0.775452	1.062132	2007	1.772436	0.855447	1.076431
1993	1.615492	0.783196	1.066135	2008	1.804726	0.860079	1.072442
1994	1.618203	0.789682	1.070240	2009	1.830522	0.864333	1.069811
1995	1.626538	0.793710	1.070954	2010	1.862533	0.867561	1.065705
1996	1.637024	0.800777	1.073267	2011	1.889022	0.871688	1.063313
1997	1.659629	0.803611	1.069424	2012	1.915065	0.879422	1.062993
1998	1.684771	0.810108	1.067721	2013	1.925885	0.884852	1.063985
1999	1.705432	0.818391	1.068370	2014	1.952627	0.890595	1.062630
2000	1.724791	0.824314	1.067802	2015	1.971527	0.894998	1.061901
2001	1.730547	0.825990	1.067590	2016	1.996196	0.900463	1.060884
2002	1.729555	0.831169	1.071073	2017	2.007657	0.905397	1.061543
2003	1.729541	0.836383	1.074342	2018	2.033197	0.911227	1.060656
2004	1.734895	0.839485	1.075058	2019	2.051796	0.918611	1.061442
				**Average**	**1.785268**	**0.842690**	**1.067250**

### 5.4 Scenario analysis

To indicate the impact of transport energy consumption and CO_2es_ reduction of substitutability between energy and capital investment in Bangladesh’s transport sector. Following Lin and Raza [52], energy-conserving and CO_2es_ reduction potential are estimated under various scenarios, as demonstrated in Table 6. As for practical implications, we have further increased the investment scenarios in energy and capital-saving technologies by 5% and 10% from 2017 to 2019. The entries in Table 6 in 2017–2019 show that a 5% rise in the capital stock of investment in energy-saving would lessen energy utilization by 4.0190, 4.3801 and 4.2497 Mtoe. As a consequence of energy-savings, the CO_2es_ reduction would reduce by 3653, 3650 and 3541 MtCO_2_. On the other hand, a 10% rise in capital stock investment in energy-saving would lessen energy usage by 4.2104, 4.5887 and 4.4520 Mtoe. As a consequence of energy-savings, the CO_2es_ would decline by 0.3827, 0.3823 and 0.3710 MtCO_2_ during 2017–2019. This emphasizes the efficiency of Bangladesh’s transport sector to obtain energy-saving and carbon reduction. Because of little variation within energy-savings and CO_2es_ reduction (see Table 6), findings in energy-saving technologies in the transport sector are much necessary for skilled labor. Moreover, this sector usually depends on fossil fuels, a growing labor force, providing business services, and rising pollution emissions. Finally, the growth of capital or investment in modernization will play an imperative role in the favor of the country’s economic wealth.

**Table 6:** Energy-saving and CO_2es_ reduction of Bangladesh’s transport sector under current scenarios (2017–2019).

Year	Scenario 1: Capital raised by 5%		Scenario 2: Capital raised by 10%	
	Energy-saving (Mtoe)	CO_2es_ reduction (Mt CO_2_)	Energy-saving (Mtoe)	CO_2es_ reduction (Mt CO_2_)
2017	4.019081	0.365371	4.210466	0.382770
2018	4.380141	0.365012	4.588719	0.382393
2019	4.249706	0.354142	4.452073	0.371006

### 5.5 Relative differences in technological progress

Technological progress in emerging countries enhances sustainable environmental output by encouraging investors to apply advanced technologies [75]. Similarly, emerging countries should concentrate on utilizing advanced technologies to distinguish the long-run association between economic development and the environment [71]. However, the technologies aim to decline CO_2es_ and enhance productivity either using human, natural resources and energy use. An effort has been done to estimate the relative differences in the technological progress of each pair of inputs in the current study. It was achieved using the aggregate trans-log production function of Bangladesh’s inter-factor and inter-fuel possibilities, linking the output elasticities and investigating coefficients from Eq. (1). The certain equation used for estimating technological progress (tp) is as shadows in Eq. (15):

(15) $$tp_{ij}=\frac{\alpha_{i}}{\varphi_{i}}-\frac{\alpha_{j}}{\varphi_{j}}$$

where $tp_{ij}$ shows the technological progress between factors i and j. $\alpha_{i}$, $\alpha_{j}$, $\varphi_{i}$, and $\varphi_{j}$ are the coefficients and output states of technical knowledge i and j. Based on the rule of thumb and researchers’ findings, which show the relevant results [38,40,58]. The assumptions show that if the $tp_{ij}$ is positive, then i is faster than j; if $tp_{ij}$ results are negative, then j is faster than i factor and if $tp_{ij}$ value is ‘0’, then there is no technical change between factors.

Based on Eq. (15) and the graphical representation form of pairs of factors (see Fig. 8), there is only a modest difference in the $tp_{ij}$ of all the input factors. As shown in Fig. 8, all the inputs, such as capital-labor, capital-transport energy consumption and labor-transport energy consumption show a positive change in technical development. It also confirms that the entire factor a value is more than zero, presenting that $tp_{ij}$ is the key is input-driven and changes between 0.009% and 0.14%. Except for capital-labor, the $tp_{ij}$ of capital-transport energy consumption and labor-transport energy consumption are a little stable over the studied period. Generally, our outcomes suggest that the relative difference in $tp_{ij}$ of labor-transport energy consumption is quicker than capital-labor and labor-transport energy consumption. This is because Bangladesh signed several agreements related to energy projects with China and investment in fuel substitution [8]. However, transport production agrees on energy technology in consideration of reducing the CO2_es_ and cost because the country has signed oil-gas agreements with various countries, including the United States, Qatar, gas transmission and development projects between Asian Development Bank, and various gas companies. Thus, energy consumption in this sector is a key issue, which can give more employment in the future for economic enhancement.

## 6 Conclusion and policy suggestions

### 6.1 Conclusion

The present study analyzes the inter-factor and inter-fuel substitution between labor, capital and energy consumption in the transport sector of Bangladesh. This research applied time-series data from 1990 to 2019 using the trans-log production function method. Due to the multicollinearity issue in the data, we employed ridge regression to control the phenomenon. The results show that:

1. We have calculated the degree of individual factors output elasticity, which shows that the entire factor (i.e., labor, capital and energy) are raising the return to scale. Although, being an under-developing nation, the output of each factor is slightly moderate, which presents a potential for Bangladesh’s transport sector, particularly in investment and energy-conservation.
2. The study analyzes the elasticity of substitution between a pair of factors’, i.e., capital-labor, capital-energy and labor-energy of the transport sector. This shows that all the pairs provide significantly increasing outcomes, with averagely of 1.78, 0.84 and 1.06 indicating that each pair in the study are a substitute. As the output grows in which energy presents a higher degree of responsiveness, flowed by labor and capital. The maximum proportion of labor-capital and labor and energy would be more appropriate for the country’s development. To see the average tests between these factors, all tests rejected the null hypothesis and the average energy is about one at the level. This proposes comparatively higher substitution potential between the pairs of factors. These results are also consistent with Bangladesh Economic Review [8], the huge investment in transportation, technology and energy-saving is proof of increasing capital, labor and energy consumption in Bangladesh. Consequently, the enhancement between factors is due to the adoption of new technologies, skilled labor and energy-conserving technologies by capital investments, which are the key sources of mitigating CO_2es_ and reducing fossil fuels.
3. For the conservation of energy and carbon emissions reduction in the transport sector, the current scenarios show that the substitution elasticity between capital investment and energy presents positive efficiency of energy-savings and CO_2es_ reduction, including the higher investment in energy-related technologies. This will advantage energy-saving, living standards, lessen costs, and increase employment. This suggests that there is a growth in technology and energy, which will reduce subsidies for growth capital.
4. Finally, the relative difference in technological progress is majorly input-driven and seems quite gradual changing between 1% and 14%. Overall, the progression between factors is mixed, in which labor-energy is faster than the other pairs of factors. This endorses that energy investment speed is faster than labor and capital. Moreover, a strong convergence between factors is shown, which may enhance and control each factor in the future.

### 6.2 Policy suggestions

The significant policies, particularly in the framework of global protest for CO_2e_ reduction and enhancing energy security are provided.

(a) Bangladesh finds itself in a state of plight, and it has to govern its future energy policy rightly. Organizing Bangladesh’s transport sector, the maximum oil consuming sector and the third CO_2es_ after the industrial and power sector can play an important role. In respect of the highest substitution elasticity between $\sigma_{K.L}$ and $\sigma_{K.TEC}$ and its trend employ that the transport sector has enhanced in a labor-intensive way due to the extra labor force and a comparatively small level of transport technology. With longer slow output, the labor force in the transport sector is declining compared with capital and energy; therefore, technical investment brings the modernization and mechanization of the transport sector. Thus, the substitution between $\sigma_{K.TEC}$ is effective in future productivity, ultimately leading Bangladesh transportation from labor-capital intensive.
(b) The elasticity of substitution between $\sigma_{K.TEC}$ demonstrates the most competing factor, indicating the most critical for adopting energy-conserving technologies by capital purchases. This will not only save energy but also mitigate CO_2es_ [18,52]. Using this substitution, the current scenario analysis proposes higher investment in energy-saving technology. This also presents that there is a potential for energy-saving and the subsequent carbon emissions of the transport sector. For this, the Government of Bangladesh is going to invest in generating energy from coal, liquefied natural gas, dual-fuel, nuclear, and renewable energy along with establishing gas and liquefied fuel-based power plants [8]. In addition, under the Memorandum of Association (MoU) between China and Bangladesh on 21-October-2012, as a consequence, objectives are concerned to uplift trade, electricity generation, transmission, distribution, energy efficiency, and renewable energy technologies. Therefore, Bangladesh will have a surplus of these technologies to save energy and reduce oil import and pollution. Finally, Bangladesh’s transport sector should follow relevant policies of developed countries, such as Muratori et al. [57] for the United States who investigated the behaviors of drivers, types of vehicles, distribution level, and future planning for measuring the impact of plug-in electric vehicles on the grid, and Lin and Xie [37] for China’s transport sector, who investigated the energy and capital investment in the transport sector to favor the economy and friendly environment.

Finally, the study is not without limitations. (i) The online availability of statistics should be made sure in the future. (ii) One of the limitations of this is that this model only concentrates on the overall transport, energy, capital, and labor for Bangladesh as a whole. However, the proposed analysis is generic and can be used for any other transport, which is creating more pollution, for example, road transport or air transport and transport link with the public. (iii) Moreover, the causal relationship and co-existence between the variables can be explored in the future. Finally, further study can be made on the accessibility of transportation’s labor, capital, trade transport and non-registered transport companies statistics is also our limitations for future study.

## Author contribution statement

Muhammad Yousaf Raza: Conceived and designed the experiments; Performed the experiments; Analyzed and interpreted the data; Contributed reagents, materials, analysis tools or data; Wrote the paper.

## Funding statement

This paper is supported by National Natural Science Fund of China (Grant No. 21BJY113).

## Data availability statement

Data will be made available on request.

## Declaration of interest’s statement

The authors declare no conflict of interest.
